# Performance assessment for EEG-based neonatal seizure detectors

**DOI:** 10.1016/j.clinph.2010.06.035

**Published:** 2011-03

**Authors:** A. Temko, E. Thomas, W. Marnane, G. Lightbody, G.B. Boylan

**Affiliations:** a Neonatal Brain Research Group, University College Cork, Ireland; b Department of Electrical and Electronic Engineering, University College Cork, Ireland; c Department of Paediatrics and Child Health, University College Cork, Ireland

**Keywords:** Performance assessment, Metrics, Neonatal EEG, Automated seizure detection, Machine learning, Support Vector Machines

## Abstract

**Objective:**

This study discusses an appropriate framework to measure system performance for the task of neonatal seizure detection using EEG. The framework is used to present an extended overview of a multi-channel patient-independent neonatal seizure detection system based on the Support Vector Machine (SVM) classifier.

**Methods:**

The appropriate framework for performance assessment of neonatal seizure detectors is discussed in terms of metrics, experimental setups, and testing protocols. The neonatal seizure detection system is evaluated in this framework. Several epoch-based and event-based metrics are calculated and curves of performance are reported. A new metric to measure the average duration of a false detection is proposed to accompany the event-based metrics. A machine learning algorithm (SVM) is used as a classifier to discriminate between seizure and non-seizure EEG epochs. Two post-processing steps proposed to increase temporal precision and robustness of the system are investigated and their influence on various metrics is shown. The resulting system is validated on a large clinical dataset of 267 h.

**Results:**

In this paper, it is shown how a complete set of metrics and a specific testing protocol are necessary to extensively describe neonatal seizure detection systems, objectively assess their performance and enable comparison with existing alternatives. The developed system currently represents the best published performance to date with an ROC area of 96.3%. The sensitivity and specificity were ∼90% at the equal error rate point. The system was able to achieve an average good detection rate of ∼89% at a cost of 1 false detection per hour with an average false detection duration of 2.7 min.

**Conclusions:**

It is shown that to accurately assess the performance of EEG-based neonatal seizure detectors and to facilitate comparison with existing alternatives, several metrics should be reported and a specific testing protocol should be followed. It is also shown that reporting only event-based metrics can be misleading as they do not always reflect the true performance of the system.

**Significance:**

This is the first study to present a thorough method for performance assessment of EEG-based seizure detection systems. The evaluated SVM-based seizure detection system can greatly assist clinical staff, in a neonatal intensive care unit, to interpret the EEG.

## 1 Introduction

Seizures are the most common neurological emergency in the neonatal intensive care unit (NICU) and are a worrying sign for medical staff and parents. The vast majority of neonatal seizures are subclinical making them difficult to detect and treat (Boylan et al., 2002; Clancy et al., 1988; Murray et al., 2008). The only available method to detect all seizures in babies is to use continuous EEG monitoring. Most hospitals lack the special expertise required to interpret EEG especially on a 24/7 basis and seizures often remain undiagnosed. A system, therefore, that could automatically detect and annotate electrographic neonatal seizures would be extremely useful to medical staff in the NICU.

A number of methods and algorithms have been proposed previously in an attempt to automatically detect neonatal seizures. However, their transition to the real-life usage in NICUs has been limited mainly by unsatisfactory performance (Faul et al., 2005). One algorithm has been implemented in an EEG monitor to date and recent results indicate a seizure detection rate of 65% (Navakatikyan et al., 2006; van Rooij et al., 2010). Further development of an automated solution to this important clinical problem is complicated by the lack of a standardised performance assessment framework. This fact perplexes a developer and complicates the extraction of promising research directions from previous published works. As a consequence, every novel neonatal seizure detection system cannot inherit the advantages of the existing alternatives, so that, each new idea is heralded as yielding the best up-to-date results (see chronologically Liu et al., 1992; Gotman et al., 1997; Celka and Colditz, 2002; Navakatikyan et al., 2006; Aarabi et al., 2007; Deburchgraeve et al., 2008; Mitra et al., 2009).

One of the main constituent parts of the standardised performance assessment framework are the metrics employed. The metrics used to report the results of these seizure detection systems vary from publication to publication. Some papers only report clinically motivated event-based metrics (Gotman et al., 1997; Deburchgraeve et al., 2008; Mitra et al., 2009); others only report epoch-based metrics (Liu et al., 1992; Aarabi et al., 2007). This fact significantly complicates the comparison of the proposed approaches. It in turn weakens the conclusions about the usefulness or efficiency of the applied techniques. Apart from different terms used to name the same metrics across the literature, the comparison of the reported systems is further complicated when a pair of metric values is reported (Gotman et al., 1997; Deburchgraeve et al., 2008; Liu et al., 1992; Aarabi et al., 2007; Navakatikyan et al., 2006; Mitra et al., 2009) rather than a complete curve of performance of the system (Greene et al., 2008; Temko et al., 2011).

Apart from metrics, the above-mentioned published studies differ in many other ways: some studies report results as an average over training and testing data (Aarabi et al., 2007) in contrast to reporting results obtained on testing data only (e.g. Temko et al., 2011; Mitra et al., 2009; Greene et al., 2008; Navakatikyan et al., 2006). Some report results by averaging over sick and healthy babies (Navakatikyan et al., 2006) in contrast to reporting results separately for each category (Mitra et al., 2009; Deburchgraeve et al., 2008). Some assess the performance based on a heuristically derived static data division to training and testing datasets (Mitra et al., 2009; Navakatikyan et al., 2006; Aarabi et al., 2007), others perform statistical tests by dividing the data repeatedly to training and testing (Temko et al., 2011; Greene et al., 2008), while others do not have separate testing data at all and report results over the data on which the algorithm was developed (Deburchgraeve et al., 2008). Last but not least, not all studies (Mitra et al., 2009) clearly report the complete etiology and gestational age (GA) of the babies used in the dataset and do not include seizure etiology e.g. hypoxic ischemic encephalopathy or whether the dataset consists of full-term or pre-term babies – this complicates the selection of the best existing algorithm for a designated group of patients in NICU.

In Temko et al. (2011), the SVM-based seizure detection system was presented and its performance was determined on a large dataset of babies with seizures. This work also investigated the main sources of misclassification in terms of most common reasons for non-detected seizures and false detections. This paper proposes a number of different metrics for the system presented in Temko et al. (2011). It is possible to display the detailed behaviour of the system in terms of these metrics. An extended analysis of the influence of the post-processing parameters on various metrics is also presented. Moreover, various ways for performance assessment that are employed in the literature of neonatal seizure detectors are outlined. This allows a better quantitative comparison of the proposed system to several existing alternatives. It is shown that reporting only the two most common event-based metrics can be misleading and a new metric is proposed to accompany the event-based metrics.

## 2 Methods

### 2.1 Performance measurements

The metrics used to assess the performance of a seizure detector task can be divided into epoch-based and event-based metrics.

#### 2.1.1 Epoch-based metrics

Epoch-based metrics can be viewed as application irrelevant metrics – every epoch is considered as a separate testing example regardless of the importance that its (in)correct classification has for a particular task. In a binary decision problem such as the seizure detection, the decision made by the classifier can be represented in a structure known as a confusion matrix or contingency table. The confusion matrix has four categories: true positives (TP) are epochs correctly labelled as seizures; false positives (FP) refer to epochs incorrectly labelled as seizure; true negatives (TN) correspond to correctly labelled non-seizure epochs and finally, false negatives (FN) refer to epochs incorrectly labelled as non-seizure.

Epoch-based metrics for seizure detection come from two theories: signal detection theory and information retrieval theory. From the former, sensitivity and specificity are reported in most papers (Liu et al., 1992; Aarabi et al., 2007; Navakatikyan et al., 2006) and are defined as TP/(TP + FN) and TN/(TN + FP), i.e. the accuracy of each class separately. When evaluating binary decision problems it is very difficult to compare the performance of various systems when only a pair of values (sensitivity and specificity) is reported. This is usually the case with systems based on a number of heuristic rules and thresholds (Liu et al., 1992; Aarabi et al., 2007; Navakatikyan et al., 2006). Provost et al. recommended the use of Receiver Operator Characteristic (ROC) curves, which show how the sensitivity of a classifier can be traded against its specificity (Provost et al., 1998). The area under the ROC curve is an effective way of comparing the performance of different systems. A random discrimination will give an area of 0.5 under the curve while perfect discrimination between classes will give unity area under the ROC curve.

ROC curves, however, can present an overly optimistic view of an algorithm’s performance if there is a large skew in the class distribution (Davis and Goadrich, 2006). This unfortunately is usually the case in seizure detection where non-seizure prevails. Precision–recall (PR) curves, which are often used in information retrieval (Manning and Schutze, 1999), have been cited as an alternative to ROC curves. While recall is the same as sensitivity, precision (also known in seizure detection literature as selectivity, relative specificity, positive predictive value (Aarabi et al., 2007)) is defined as TP/(TP + FP), i.e. a percentage of correctly produced/predicted seizure epochs. Unlike the ROC area, the PR area is not equal to 0.5 for random discrimination but depends on class priors, that is, the number of datapoints in each class. The unity PR area indicates perfect discrimination. Only a few papers report the ROC curves for their algorithms (Greene et al., 2008) and none have reported the PR curve.

#### 2.1.2 Event-based metrics

The event-based metrics are thought to reflect the performance of a system for a specific application. Unlike the epoch-based metrics, the subsequent decisions of the same class are joined to create an event. Two scores are defined; good detection rate (GDR) is the percentage of seizure events correctly identified by the system as labelled by an expert in neonatal EEG. If a seizure was detected any time between the start and end of a labelled seizure this was considered a good detection (Gotman et al., 1997).

The other score is the number of false detections per hour (FD/h) calculated as the number of predicted seizure events in 1 h that have no overlap with actual reference seizures. To cope with the spiky nature of false detections, the metric FD/h is at times reported by joining not only subsequent false detections but also those that lie closer than 30s apart from each other (Gotman et al., 1997). The resulting metric is always better than the initially defined FD/h and is marked FD/h (30s) throughout this work.

The curve of variation of GDR with FD/h should be reported to enable a valid comparison of different systems. To the best of our knowledge, this has not been reported previously. The main reason for this is that many algorithms require the careful selection of a number of rules and thresholds (Gotman et al., 1997; Deburchgraeve et al., 2008; Mitra et al., 2009) and do not allow a continuous system output which can be then used to build performance curves.

A new metric which is proposed in this work is the mean false detection duration (MFDD). It is assessed by averaging the durations of all false detections produced by the system at a single operating point (with a chosen threshold). It will be shown in the experimental part of this paper that reporting the two event-based metrics can be misleading unless the MFDD is also reported. In a real application, FD/h indicates the number of times a clinician has to check the results of an automatic detector in vain; however, it is important, we believe, to also report the mean duration of these false detections. For instance, if both systems give 90% of GDR, the first one at a cost of 1 FD/h with 20 m duration and the other at a cost of 2 FD/h each with 1 m duration, the second system may be preferred as the results of the first system imply that ∼33% of time a clinician has to check the EEG monitor in vain, with only ∼3% of time in the second case.

### 2.2 Dataset

A dataset composed of EEG recordings from 17 newborns obtained in the NICU of Cork University Maternity Hospital, Cork, Ireland was tested. The patients were full-term babies ranging in gestational age from 39 to 42 weeks. All newborns had seizures secondary to hypoxic ischemic encephalopathy (HIE). A Viasys NicOne video EEG machine was used to record multi-channel EEG at 256 Hz using the 10–20 system of electrode placement modified for neonates. The following eight bipolar EEG channels are used in this study: F4-C4, C4-O2, F3-C3, C3-O1, T4--C4, C4-Cz, Cz-C3, and C3-T3. The combined length of the recordings totals 267.9 h (mean duration per patient is 15.76 h) and contains 705 seizures which range from less than 1 min to more than 10 min in duration (mean seizure duration is 3.89 min). The dataset contained a wide variety of seizure types including both electrographic-only and electro-clinical seizures of focal, multi-focal and generalized types. All seizures were annotated independently by 2 experienced neonatal electroencephalographers using video EEG. The continuous EEG recordings were not edited to remove the large variety of artifacts and poorly conditioned signals that are commonly encountered in the real-world NICU environment. The dataset used is thoroughly described in Temko et al. (2011).

### 2.3 Overview of the SVM-based seizure detector

**Fig. 1:**
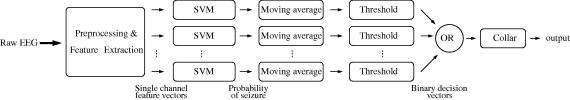
Architecture of the SVM-based seizure detection system.

The system described in detail in Temko et al. (2011) is shown in Fig. 1. The EEG is down-sampled from 256 to 32 Hz with an anti-aliasing filter set at 12.8 Hz. The EEG is then split into 8s epochs with 50% overlap between epochs. Fifty-five features are extracted which represent both time and frequency domain characteristics as well as information theory based parameters. The features extracted from each epoch are then fed to train one SVM classifier.

Classification consists of two steps – training and testing. The leave-one-out (LOO) cross-validation method is used to assess the performance of the system for patient-independent seizure detection. This way, all but one patients’ data is used for training and the remaining patient’s data is used for testing. This procedure is repeated until each patient has been a test subject and the mean result is reported. The leave-one-out method is known to be an almost unbiased estimation of the true generalization error (Vapnik, 1982); that is the performance reported with the leave-one-out method is the most similar to the performance this system would show on all unseen data of infinite size once it is trained on all available data. In Section 4 of this paper, various alternatives to the chosen scheme are reviewed.

The training data for the SVM classifier are first normalized anisotropically by subtracting the mean and dividing by standard deviation to assure commensurability of the various features. This normalizing template is then applied to the testing data. In the testing stage, the obtained classifier is applied separately to each channel of the testing data. The outputs of the SVM are converted to posterior probabilities and filtered with a moving average filter (MAF). The averaged value is then compared to a threshold from the interval [0 1]. After comparison, binary decisions are taken per channel: 1 for seizure and 0 for non-seizure. The binary decisions are then fused as follows: if there is a seizure in at least one channel, the whole epoch is marked as a seizure, otherwise it is denoted as a non-seizure. The ‘collar’ technique is applied last – every seizure decision is extended from either side to compensate for possible difficulties in detecting pre-seizure and post-seizure parts. In the experimental part of this study, the influence of the collar on the system performance, especially for event-based metrics is analyzed.

## 3 Results

An example of how reporting various metrics may be used to assess the performance of the system and to provide a detailed overview of the system behaviour is given in this section.

**Fig. 2:**
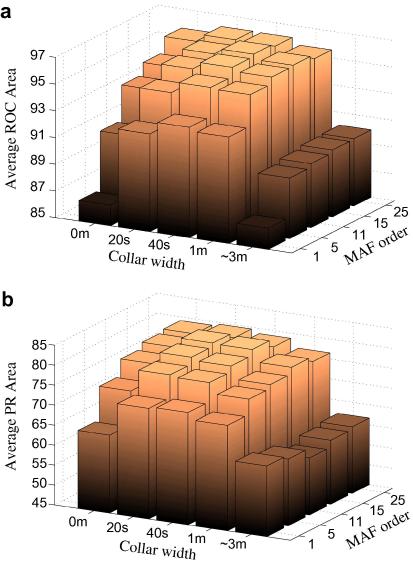
The average ROC (a) and PR (b) areas of the SVM-based system for different values of MAF and collar.

The performance of the system in terms of ROC and PR areas for various parameters of post-processing is shown in Fig. 2. It can be seen that the best performance is achieved with MAF equal to 15 epochs and collar equal to 40s in terms of both ROC and PR areas.

**Fig. 3:**
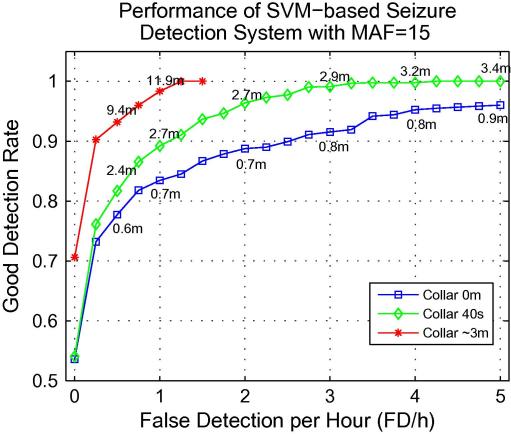
The influence of the collar width on the GDR against FD/h curve. MFDD is shown in minutes for 0.5, 1, 2, 3, 4, and 5 FD/h.

The effect of post-processing is further explored on event-based metrics. Specifically, widths of 0, 40s (10 epochs) and ∼3 m (50 epochs) were chosen to investigate the influence of the collar on the event-based metrics. The curve of variation of GDR with FD/h for 8 different collar widths with MAF = 15 is shown in Fig. 3. The figure indicates that the event-based metrics alone can be made arbitrary good using the collar post-processing step.

**Fig. 4:**
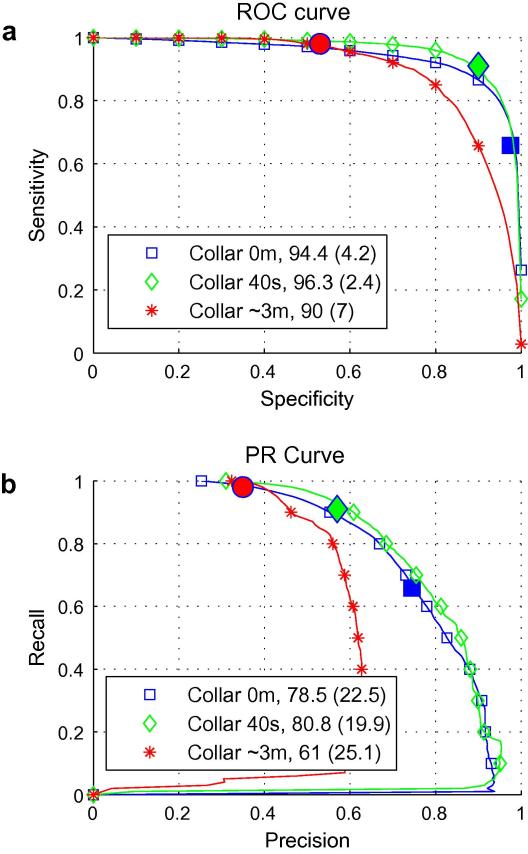
The ROC (a) and PR (b) curves of the SVM-based system at MAF = 15 for various widths of collar. The highlighted points indicate the performance of the system at 1 FD/h.

Fig. 4 shows ROC curves (a) and PR curves (b) for the three different collar widths along with the resulting mean and standard deviation of the area under each curve in %. The three highlighted points on each curve correspond to the system performance at 1 FD/h (Fig. 3) which is achieved by setting the threshold on probability of seizure to 0.6, 0.4, and 0.2 for no-collar, 40s collar and 3 m collar systems, respectively. Thus, for a 40s collar, at 1 FD/h, sensitivity of 90%, specificity of 90%, and GDR of 89% can be achieved.

Table 1 shows the ROC area, PR area, GDR and sensitivity at 0.5 FD/h and at 1 FD/h for each patient in the database.

**Fig. 5:**
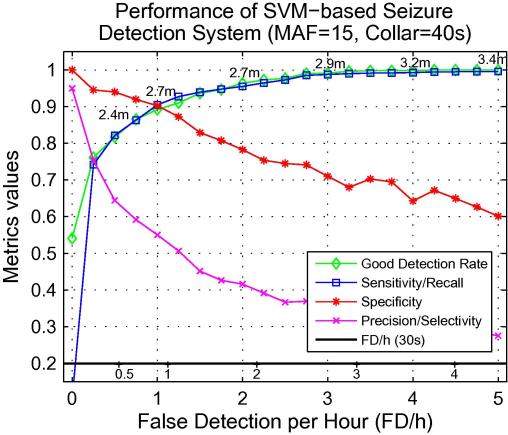
Summary of the epoch-based and event-based metrics mapped at the common *x*-axis of FD/h.

To better examine the behaviour of the system with MAF = 15 and collar = 40s, results are shown in Fig. 5 where various epoch-based and event-based metric values are mapped on the common FD/h *x*-axis. The figure can be used to describe fully the performance of the system and for a complete comparison among the various seizure detection systems.

## 4 Discussion

### 4.1 Post-processing parameters and their influence on the system performance

As can be seen from Fig. 2, the impact of the post-processing steps on the system performance is significant. Without post-processing (MAF = 1, collar = 0s), the ROC area is 86.5% (±6.8). Applying the MAF on its own, the ROC can be increased to 95.5% (±3.3), using a 25-epoch filter. Similarly, using just the collar technique, the ROC can be increased to 93.3% (±4.6). The largest overall ROC (96.3 ± 2.4) is obtained using MAF = 15 and collar = 40s. The increase in PR area shown in Fig. 2b is also meaningful – from 64.6% (±27.2) without post-processing to 80.8 (±19.9) for MAF = 15 and collar = 40s. Both figures suggest that with MAF in the range 5–15 and with the collar around 40s, the system performs almost equally well. In fact, the same seizure detection system has been applied to an adult database of intracranial EEG in Faul et al. (2009), and the best ROC area of 94.1% has been obtained with MAF = 15 and collar = 32s. This indicates that there is a need to compensate for difficulties in correctly classifying sections of the EEG immediately prior to and after a seizure event. Alternatively, if the collar of half the length of MAF is applied, the decisions are extended to the actual start and end of the processed data. That is, with MAF equal to 15 epochs, 7 epochs before and 7 epochs after the current epoch are used to calculate the probability of seizure for the current epoch. If the epoch is finally marked as seizure, the collar of 7 epochs (28s) will extend the seizure decision to actually match the real beginning and ending points of the processed data. Consequently, in our case, with MAF = 15 and collar = 40s, only 12s (40–28) can be considered as the required compensation for non-detected pre- and post-seizure parts.

It is also evident from Fig. 2b that the average PR areas are significantly smaller than the average ROC areas. This is due to the fact that the ROC area does not take into account the priors of each class (seizure and non-seizure) while the PR area is affected by the data imbalance presented in seizure detection problems. As it is shown in per patient results below, the PR areas become more representative of actual performance than the ROC areas for patients with large data imbalance.

### 4.2 Event-based metrics and MFDD

It is obvious from Fig. 3, that if a constant GDR is specified, then the FD/h can be decreased by increasing the collar width: the system with the largest collar reaches 90% of GDR with ∼0.25 FD/h, the system with collar of 40s yields 90% of GDR with ∼1 FD/h, and the system with no-collar – with ∼2.5 FD/h. It is worth noting that the larger the choice of collar the greater the tendency for short sequences of false detections to be joined together to produce one single false detection. It is therefore apparent that both event-based metrics can be made arbitrarily good by increasing the collar width and thus reporting only GDR and FD/h can be misleading. In this situation, the proposed MFDD metric may be useful. Fig. 3 shows that the system with largest collar can obtain GDR of ∼98% with a cost of having 1 FD/h lasting on average ∼12 m. In comparison, the no-collar system has consistently lower values of GDR (∼83% at 1 FD/h) but also the mean duration of a false detection is considerably lower (∼0.7 m in comparison to ∼12 m). The system performance with the collar width equal to 40s falls between these. The results in Fig. 3 also indicate that, since the event-based metrics can be made arbitrarily good using the collar post-processing step, then they should not be the metrics used to optimise the system design.

### 4.3 ROC and PR curves

It can be seen from Fig. 4a, that despite having a high GDR, the widest collar results in the lowest specificity (0.53) at 1 FD/h. In this case therefore, only slightly more than half of all non-seizure segments are correctly detected by that system. On the contrary, the no-collar system classifies correctly almost all non-seizure epochs (high specificity) but only around 65% of seizure epochs are identified. Obviously, it would be impossible to compare these two systems if only the outlined points were reported. However, the comparison is much easier if the curves of performance are reported instead. The smallest standard deviation of the ROC area (2.4%) for the 40s-collar system indicates that the system is the most stable, performing equally well for all the patients in the database.

In contrast to the low values of specificity obtained for 1 FD/h in Fig. 4a, the low value of precision for the ∼3 m-collar system in Fig. 4b at the highlighted point indicates that less than 40% of all produced seizure epochs are indeed seizures. However, as only 1 FD/h is produced at this point with almost 100% of recall, then most falsely-detected seizure epochs are actually concatenated to detected seizures. We can also see that unlike ROC curves, the PR curves of all systems indicate there is still large room for improvement which can be achieved by increasing the temporal precision of the system.

### 4.4 Per patient results

For all the patients shown in Table 1, the ROC areas obtained are larger than 91%. In comparison, the PR areas differ significantly, which is reflected in the large standard deviation shown in Fig. 4b. According to the high ROC areas of every patient, it might be expected that the system obtains high GDR for all of them. It might be also expected that allowing an increase in FD/h will produce a commensurate improvement in GDR. Indeed, in four out of 17 patients the classifier achieves a GDR of 100% at 0.5 FD/h. This can be increased to six patients with a false detection rate of 1 FD/h. On average, 40% relative improvement is achieved for GDR when changing the operating point from 0.5 to 1 FD/h.

However, for patients 1, 2, 5, 7, and 10, which are highlighted in Table 1, the GDRs at 0.5 FD/h are lower. Additionally, for patients 1, 2, and 10 there is no increase in GDRs even when 1 FD/h is allowed, and for patient 7 the increase is marginal. In fact, the amount of time when patients 1, 2, and 7 are having seizures is less than 2% of the total time (Table 1). Because of this huge data imbalance, for these patients, the PR areas are more representative of actual performance and low GDRs are reflected in the PR areas rather than in the ROC areas.

We and others have reported the discrepancy that exists between seizure number and actual seizure burden, i.e. the total amount of time the baby spends in seizure (Murray et al., 2008). Therefore for our seizure detection work we have been keen to express seizure burden as well as the number of seizure events when reporting system performance. The sensitivity metric given in Table 1 shows the percentage of seizure burden detected in every patient at 0.5 and 1 FD/h operating points. It can be seen that in general the average sensitivity and the average GDR are quite similar for each operating point: GDR of 82.7 vs. sensitivity of 82.7 for 0.5 FD/h, and GDR of 89.2 vs. sensitivity of 90 for 1 FD/h. The sensitivity metric shows that even for patients 1, 2, and 7 with lowest amounts of seizure burden in our dataset, the algorithm still detects a total seizure burden of ∼15, ∼12, and ∼4 m at 0.5 FD/h, respectively. These can be increased to ∼17, ∼14, and ∼5 m with a false detection rate of 1 FD/h. For patient 5, whose sensitivity indicates that only 30.3% of seizure burden is detected at 0.5 FD/h, the algorithm still correctly detects approximately 86 m of seizure activity and can be increased to ∼245 m with a false detection rate of 1 FD/h. It can be seen that the lowest PR areas are produced for the patients where the lowest seizure burden was detected, that is, for patients 1, 2, and 7. Thus, this metric can be indicative of the amount of seizure burden detected.

A proper investigation of how seizure morphology and location can influence the seizure detection rate and detected seizure burden is required and will be the focus of our future work.

### 4.5 The complete performance of the system on one graph

As it can be seen from Fig. 5, the system can correctly detect ∼89% of seizure events with a cost of 1 FD/h with an average duration of 2.7 m, ∼96% with a cost of 2 FD/h each with an average duration of 2.7 m, or ∼100% with a cost of 4 FD/h each of average duration of 3.2 m.

For a false detection rate in excess of 0.25 FD/h, the event-based GDR tends to closely match the epoch-based sensitivity/recall measure which also measures the amount of detected seizure burden. This indicates that the system shows equally high temporal precision and detection rates. The main significant difference appears at 0 FD/h where a sensitivity of less than 20% results in a GDR in excess of 50%. At this point, only the most evident seizure parts of more than a half of all seizure are detected with no false detections produced.

The robustness of the system can also be observed by examining the FD/h (30s) metric which appears to be quite close to the actual FD/h up to 4 FD/h. Hence, for the proposed system there is no need to adapt the metric to better match the system behaviour. It is worth outlining the difference between the collar technique and the FD/h (30s) metric as both approaches are shown to decrease the actual FD/h. In the first case, the collar forms a part of the overall classification system and is applied before any metrics are calculated. Thus, increasing the collar will decrease FD/h but it also influences other metrics as shown in Fig. 4. Here the largest collar resulted in the lowest ROC and PR areas. In the second case, the initial metric FD/h is changed to FD/h (30s) to count smaller number of false detections.

In fact, Fig. 5 shows the entire performance of the system in terms of the epoch-based and event-based metrics. This is the key figure which should be provided by any system to enable a fair comparison. From Fig. 5, it is possible to define a single evaluation metric for a complete comparison (for instance, in evaluation campaigns, etc.), such as, for example, a (weighted) average of the areas under the GDR, sensitivity, specificity, and precision curves up to 3 FD/h.

A variety of metrics were calculated in this study, however, the post-processing parameters of the system were chosen to maximize the ROC area, with the other metrics observed to help explain the behaviour of the system in a particular situation or for a chosen patient.

### 4.6 Comparison with recently reported systems

Most recently published neonatal seizure detection systems claim to offer the best “state of the art” performance. However, the testing protocol used in the reported papers is so different that such claims are difficult to verify. A fair comparison among seizure detection systems is quite complicated for several objective reasons which are outlined below.

Consider first the data used to test the systems. Several studies do not have a clear division of the data used to train and test the algorithm and thus, in many cases, report test results on the data on which the system has been developed (Deburchgraeve et al., 2008). There are numerous papers in the machine learning literature including neonatal seizure detection (Gotman et al., 1997) that shows that the performance obtained on the development data is significantly better than the performance obtained on unseen testing data. In general, the performance on the development data is proportional to the number of degrees of freedom of corresponding classification system – that is, for example, the number of rules and thresholds used in Deburchgraeve et al. (2008). In other studies, the testing data is allocated but they still report results by averaging over training and testing data which similarly leads to a significantly over-optimistic performance assessment (Aarabi et al., 2007).

The majority of studies on neonatal seizure detection have performed a split of data into training and testing, either performed once or repeatedly in a certain statistical routine. Most studies prefer a static data division (also known as the hold-out or split sample method (Kohavi, 1995; Molinaro et al., 2005) – one fixed partition of the available data for a training and a separate testing set. The main advantage of the static data division is that it is extremely simple and computationally cheap – results can be obtained after a single experimental run. The static data division is often used in the machine learning community for comparison of various decision makers on a common dataset (Guyon et al., 2006) but its usage is discouraged for performance assessment as it has several major disadvantages. First, it results in a very inefficient data usage – a section of the data is never exploited in training and the other part is never used for testing. This is additionally reinforced by taking into account that the datasets for neonatal seizure detection are quite difficult to obtain and never too large. Second, such a division results in a potentially large bias. Over-optimistic or indeed over-pessimistic results can be obtained depending on what seems an “arbitrary” partition of the data – a “good” or “bad” split (Kohavi, 1995; Molinaro et al., 2005). For instance, in this paper we report an ROC of 96.3% obtained by averaging in the LOO procedure. If we perform a static data division with 75% of the whole dataset allocated for training and 25% for testing where patients 4, 11, 14, and 17 are in the testing data, we could easily report an ROC of 98.8%. Of course, in this case the test subjects were chosen to maximize the resultant average ROC area (see Table 1); however, no studies which use the static data division explain what heuristic is used to determine the data partition. In contrast, apart from the lowest possible bias, the LOO eliminates any subjectivity from the testing protocol, hence it can be repeated and exactly the same results will be obtained. The major disadvantage of LOO is that it is computationally more expensive than the static data division – one has to repeat the same experiment several times before final results are obtained. However for most neonatal seizure datasets which consist of dozens (not hundreds) of subjects, the LOO procedure is completely feasible. Once the datasets grow to hundreds of patients, the LOO can be approximated with a computationally cheaper *N*-fold cross-validation (Molinaro et al., 2005).

Another point to be taken into account is that with the static division of the dataset the performance of a particular model trained on a particular chunk of data is reported. In practice, a reader or developer who wants to exploit a reported technique will most likely have a different dataset at hand; thus, the reported performance cannot be guaranteed for him/her. What is examined with the LOO procedure is not a particular model but indeed the methodology used to obtain such a model. The last point means that the LOO estimate gives effectively a robust prediction of the performance that other researchers/practitioners will obtain using this method, but trained on their data. Only a few papers used the LOO for performance assessment (Greene et al., 2008; Temko et al., 2011).

Averaging over inhomogeneous data also affects performance assessment. In Aarabi et al. (2007) patient-independent and patient-dependent results are averaged. Apart from the fact that the practical usefulness of the patient-dependent seizure detector for neonates is limited, it has also been shown that patient-dependent seizure detection obtains significantly better results. Such averaging is clearly over-optimistic. In Navakatikyan et al. (2006), the performance is averaged over healthy and sick babies. In Mitra et al. (2009) and Temko et al. (2010b) it has been shown that neonatal seizure detectors produce significantly better performance on healthy babies than on sick babies. Thus, the averaged performance can be made arbitrarily good by increasing the amount of healthy patient data in the study. For instance, instead of reporting a GDR of 82% at 0.5 FD/h for 17 seizure babies in our current study, we could average it with 0.12 FD/h obtained with the same SVM-based seizure detector applied to a set of 47 healthy babies in Temko et al. (2010b), and report an average per patient of ∼0.22 FD/h. In fact, the large difference between the FD/h obtained on healthy babies and on sick babies suggests that the results on sick and on healthy babies should be reported separately as it has been done in Mitra et al. (2009). In a certain sense, these values indicate the average upper and lower bounds on FD/h achievable in practice.

It has been also argued in Temko et al. (2010b) that the FD/h metric can be affected by the density of seizures in a recording. Long recordings, which are most representative for the task in practice, may result in a lower density of seizures and are more inclined to produce higher number of false detections. For instance, in Mitra et al. (2009), the average number of seizures per hour was ∼4.9, in Navakatikyan et al. (2006) there were ∼4 seizures per hour, and in Deburchgraeve et al. (2008)) ∼3.3 seizures per hour. Comparing the statistics of the datasets in the mentioned studies, the results obtained on our dataset with ∼2.6 seizures per hour can be seen as an over-pessimistic performance assessment.

Different seizure etiology and gestational age of the babies in the exploited datasets is another issue which is important for comparison of detectors. Not all studies (Mitra et al., 2009) clearly report the complete seizure etiology and GA of babies in the dataset used such as, whether the seizures are secondary to e.g. hypoxic ischemic encephalopathy or whether the dataset consists of full-term or pre-term babies – it complicates the selection of the best existing algorithm for a designated group of patients in NICU.

It is worth noting that for the above-mentioned reasons, as summarised in Table 2, there are so many possible permutations of the testing protocol, experimental setup and metrics that it is quite easy to achieve a reasonably good performance and hence claim state-of-the-art results. The results reported in our work were not increased by averaging over training and testing data, not by averaging over sick and healthy patients, nor by using a heuristically chosen static data division. Additionally, the dataset contains long recordings of patients with well-defined etiology and GA, and therefore is truly representative of the real-life situation in the NICU.

**Fig. 6:**
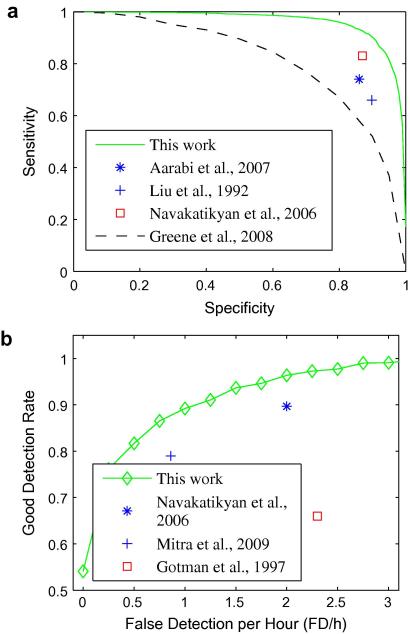
A comparison with recently reported systems using the epoch-based (a) and event-based (b) metrics.

A comparison of results with other studies is also complicated by a variety of metrics, and the way authors report their results. Below, there is an attempt to compare the algorithms for neonatal seizure detection reported in several recent studies. The comparison is only possible because the curves of performance (Fig. 5) are reported in our study. In this attempt, a point in one metric will be fixed for both systems while other metrics will be observed and compared. Given that non-seizure data prevails in the database, specificity is the most continuous metric. The continuity of specificity allows a more accurate matching of the metric values. Thus, this metric will be fixed in comparison. Additionally, Fig. 6 shows a summary of the comparison with the recently reported systems which have been tested on a relatively large datasets using the epoch-based (a) and event-based (b) metrics.

In a system which utilises a classifier based on a multilayer perceptron (Aarabi et al., 2007), a specificity of 86% and sensitivity of 74% at 1.55 FD/h has been reported. At a specificity of 86% (Fig. 5), the system reported in this study achieves a sensitivity of 93% with 1.3 FD/h. The performance obtained in Aarabi et al. (2007) is also shown in the epoch-based results in Fig. 6a.

In Navakatikyan et al. (2006), most metrics have been reported. However as the algorithm was based on a set of heuristic rules and thresholds, the metrics have been reported at a single operating point in contrast to the complete curve of performance. A specificity of 87%, sensitivity of 82.8%, precision of 48%, and GDR of 89.7% at 2 FD/h have been observed. In comparison, at a specificity of 87%, our system achieves a sensitivity of 93%, precision of 51%, and GDR of 91% at 1.25 FD/h. The results obtained in Navakatikyan et al. (2006) are also shown in Fig. 6a and b.

The system developed in Gotman et al. (1997) with GDR 71% at 2 FD/h, and the system developed in Liu et al. (1992) with a sensitivity of 65.9 and specificity of 89.8, showed significantly poorer results than those of other systems compared here as shown in Fig. 6a and b.

In Deburchgraeve et al. (2008), a GDR of 85% at 0.66 FD/h have been reported. However, as neither epoch-based metrics were calculated nor mean false detection duration, it is difficult to compare their results with ours. For example, our system with MAF = 15 and collar = ∼3 m will achieve 95% of GDR at 0.66 FD/h (Fig. 3), although we know that it will be done at the expense of longer false detection durations (∼12 min) which is not optimal for clinical use. This situation shows again that reporting only the event-based metrics can be misleading unless accompanied with MFDD or epoch-based metrics. Additionally, because no unseen test data was allocated in that study, its performance is excluded from Fig. 6b.

In Mitra et al. (2009), the GDR of 79% at 0.86 FD/h have been reported. Again, a set of heuristic rules and thresholds resulted in the single operating point (Fig. 6b). No epoch-based metrics were calculated either. However, the paper has reported the distribution of the durations of false detections from which the majority of false detections lasted between 20 and 60 s. Thus it can be roughly compared to the no-collar system presented here (in which MFDD was around 40s) which at 0.86 FD/h achieves the GDR of 83%.

Direct comparison can be made to our previous work (Greene et al., 2008) as the dataset and testing protocol are very similar to this study. The system based on a linear discriminant classifier yielded an ROC area of 82% in comparison to 96.3% obtained here (Fig. 6a). The increase in performance is due to both the utilisation of the SVM classifier and the proposed post-processing steps.

## 5 Conclusion

The study has presented an overview of common metrics, experimental setups and testing protocols forming a framework to facilitate possible future comparisons between EEG-based seizure detection systems. An SVM-based multi-channel neonatal seizure detection system has been overviewed in the proposed framework. The system has been validated on a large clinical dataset and the results have been reported. Two post-processing steps have been introduced and their influence on the system performance has been analysed. It has been shown that reporting only event-based metrics may lead to over-optimistic performance and a new metric has been proposed here to accompany the event-based metrics. The proposed SVM-based seizure detection system allows for the control of the final decision by choosing different confidence levels which in turn makes the proposed system flexible for clinical needs.

## Figures and Tables

**Table 1:** Performance of the SVM-based seizure detection system for each patient (MAF = 15, Collar = 40s).

Patient	Record length (h)	Seizure events	Mean seizure duration	Min seizure duration	Max seizure duration	ROC area	PR area	GDR@0.5 FD/h	Sens@0.5 FD/h	GDR@1 FD/h	Sens@1 FD/h
1	18.23	17	1′30′′	17′′	3′54′′	91.46	41.2	58.8	61.2	58.8	65.0
2	24.74	3	6′10′′	55′′	11′09′′	94.64	62.23	66.7	67.4	66.7	75.0
3	24.24	149	2′18′′	10′′	10′43′′	96.89	92.69	84.0	91.2	95.3	98.1
4	26.10	60	1′03′′	25′′	1′46′′	98.5	78.44	93.2	95.2	100	100
5	24	49	5′54′′	21′′	31′01′′	91.5	71.25	63.2	30.3	93.9	85.3
6	5.69	41	1′09′′	26′′	1′53′′	94.6	74.17	100	99.2	100	99.3
7	24.04	6	1′04′′	18′′	1′28′′	97.95	31.71	50	57.0	66.7	78.5
8	24.53	17	5′57′′	29′′	19′14′′	97.05	80.84	72.1	70.1	92.7	80.9
9	24.04	156	5′16′′	16′′	37′06′′	95.49	97.37	100	96.3	100	96.8
10	10.06	25	5′26′′	10′′	21′22′′	93.75	87.87	60	78.4	60	78.8
11	6.19	15	5′26′′	26′′	7′49′′	99.27	97.86	93.3	99.0	93.3	99.1
12	12	29	2′11′′	13′′	6′24′′	97.07	81.03	79.3	77.5	96.6	95.8
13	12.13	25	4′06′′	71′′	12′16′′	97.35	90.3	100	84.5	100	87.4
14	5.48	11	8′34′′	69′′	30′36′′	98.51	96.56	90	95.4	100	98.2
15	12.16	59	2′05′′	11′′	7′08′′	97.78	94.21	89.4	96.7	92.7	96.8
16	7.63	31	10′23′′	2′14′′	34′37′′	96.53	98.57	100	96.9	100	96.9
17	6.64	12	8′32′′	44′′	23′16′′	98.77	97.15	88.8	92.5	100	98.2
Mean						96.3	80.8	81.7	81.7	89.2	90

**Table 2:** Statistics of the testing dataset and testing protocols of the compared systems.

Study	Size (hours)	#Patients seiz/nonseiz	Data origin	GA/etiology	Testing protocol	Metric	Notes on results
Navakatikyan et al. (2006)	24.4	17/38	Royal Brisbane and Women’s Hospital, Royal Children’s Hospital, Brisbane	Term, pre-term/–	Static	Single values	Averaged over seizure and non-seizure babies
Aarabi et al. (2007)	86	10	North Hospital of Amiens	Term/–	Static	Single values	Averaged over training and testing datasets
Deburchgraeve et al. (2008)	217	21/5	Sophia Children’s Hospital, Rotterdam	Term/–	Static	Single values	Testing data is development data
Greene et al. (2008)	252	17	Cork University Maternity Hospital	Term/HIE	LOO	Curves	–
Mitra et al. (2009)	33.6	28/48	Texas Children’s Hospital, Heuston	–/–	Static	Single values	–
Present study	267	17	Cork University Maternity Hospital	Term/HIE	LOO	Curves	–
